# Unlocking the Pragmatic Potential of Regenerative Therapies in Heart Failure with Next-Generation Treatments

**DOI:** 10.3390/biomedicines11030915

**Published:** 2023-03-15

**Authors:** Yoshikazu Kishino, Keiichi Fukuda

**Affiliations:** Department of Cardiology, Keio University School of Medicine, Tokyo 160-8582, Japan

**Keywords:** CM, cardiomyocyte, hiPSC, human-induced pluripotent stem cell, iPSC, induced pluripotent stem cell, heart failure

## Abstract

Patients with chronic heart failure (HF) have a poor prognosis due to irreversible impairment of left ventricular function, with 5-year survival rates <60%. Despite advances in conventional medicines for HF, prognosis remains poor, and there is a need to improve treatment further. Cell-based therapies to restore the myocardium offer a pragmatic approach that provides hope for the treatment of HF. Although first-generation cell-based therapies using multipotent cells (bone marrow-derived mononuclear cells, mesenchymal stem cells, adipose-derived regenerative cells, and c-kit-positive cardiac cells) demonstrated safety in preclinical models of HF, poor engraftment rates, and a limited ability to form mature cardiomyocytes (CMs) and to couple electrically with existing CMs, meant that improvements in cardiac function in double-blind clinical trials were limited and largely attributable to paracrine effects. The next generation of stem cell therapies uses CMs derived from human embryonic stem cells or, increasingly, from human-induced pluripotent stem cells (hiPSCs). These cell therapies have shown the ability to engraft more successfully and improve electromechanical function of the heart in preclinical studies, including in non-human primates. Advances in cell culture and delivery techniques promise to further improve the engraftment and integration of hiPSC-derived CMs (hiPSC-CMs), while the use of metabolic selection to eliminate undifferentiated cells will help minimize the risk of teratomas. Clinical trials of allogeneic hiPSC-CMs in HF are now ongoing, providing hope for vast numbers of patients with few other options available.

## 1. Introduction

Heart failure (HF) represents a significant burden to patients and healthcare systems. It is estimated that HF affects approximately 60 million people worldwide [1] and is the most common cause of hospitalization in the elderly [2]. Patients with HF experience poor quality of life (QoL) [3] and have 5-year survival rates <60%, worse than many common cancers [4,5]. Moreover, the burden of HF is increasing as the population ages, as risk factors such as diabetes and obesity increase in prevalence, and as more individuals survive coronary events, such as acute myocardial infarction (AMI) [6].

Approximately 50% of HF cases occur with reduced ejection fraction (HFrEF; a left ventricular [LV] ejection fraction [LVEF] ≤ 40%) [6]. The main foundation for the treatment of HFrEF primarily comprises oral therapies (i.e., drugs), including angiotensin converting enzyme inhibitors, angiotensin receptor blockers, beta-blockers, mineralocorticoid antagonists, and more recently, angiotensin receptor-neprilysin inhibitors, sodium-glucose cotransporter 2 inhibitors, soluble guanylate cyclase stimulators, such as vericiguat, and a funny current channel inhibitor (ivabradine). Device-based approaches, such as cardiac resynchronization therapy, implantable cardioverter defibrillators, and LV assist devices, may also be used in some patients [7].

Currently available guideline-directed medical and device therapies can only act on and support residual cardiomyocytes (CMs), and prognosis remains poor for many patients. Although heart transplantation may be an option for patients with advanced HF, this approach is rarely used, partly limited by a shortage of donor organs [8]. Consequently, the ability to generate new CMs and repair the damaged myocardium represents an attractive prospect for helping to improve the prognosis of patients with HF. Cell-based therapies promise to provide patients with new fully functional CMs to repair and/or replace injured heart tissue in patients whose therapeutic options are otherwise limited.

Here, we review the progress to date in the development of cell-based therapies for HF, summarizing early clinical data from double-blind trials of first-generation multipotent cell therapies, before focusing on preclinical data and ongoing clinical trials for next-generation therapies based on human pluripotent stem cells (hPSCs).

## 2. A Brief History of Regenerative Medicine for HF

Early cell therapies aimed at treating HF have been based on multipotent cells, which are cells from tissues, such as bone marrow (BM), adult adipose tissue, or the umbilical cord (UC), that can differentiate into multiple cell types within a restricted number of lineages.

**Figure 1:**
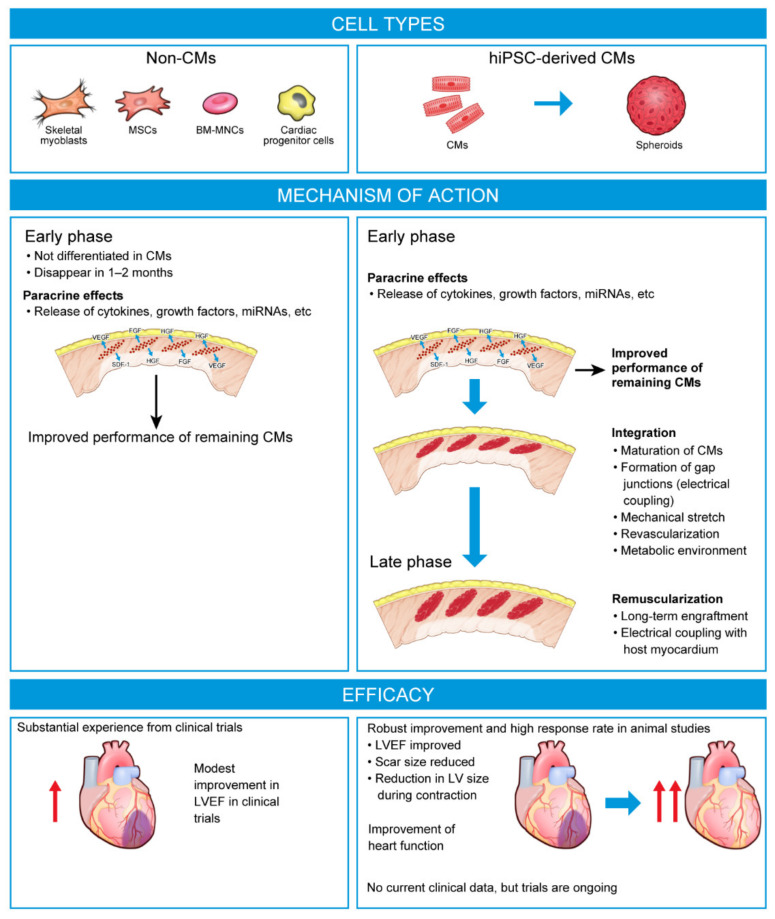
Mechanism of action of next-generation cell therapies versus first-generation cell therapies. In contrast to first-generation therapies, which are largely limited to paracrine effects, next-generation therapies aim to promote remuscularization of the heart. BM-MNC, bone marrow-derived mononuclear cell; CM, cardiomyocyte; FGF, fibroblast growth factor; HGF, hepatocyte growth factor; hiPSC-CM, human-induced pluripotent stem cell-derived cardiomyocyte; LV, left ventricle; LVEF, left ventricular ejection fraction; miRNA, micro ribonucleic acid; MSC, mesenchymal stem cell; SDF-1, stromal cell-derived factor-1; VEGF, vascular endothelial growth factor.

A multitude of preclinical studies have assessed the cardiac repair potential of multipotent cells in small and large animal models of myocardial injury. Many of these studies have shown that transplantation of multipotent cells could improve cardiac function [9,10,11]. However, engraftment rates were consistently low, with most transplanted cells quickly lost into the peripheral circulation and the cardiac benefits being moderate or transient [10,11]. The benefits of these therapies would appear to be mediated not by directly replacing the damaged myocardium, but through non-contractile, paracrine effects that help support the function of existing CMs through the release of exosomes, growth factors, and matrix metalloproteinases into the local environment, promoting angiogenesis, and reducing inflammation and fibrosis [10,11] (Figure 1). The modest benefits of these first-generation cell therapies on cardiac function in preclinical models were sufficient to encourage their assessment in double-blind clinical trials in patients following AMI or with ischemic cardiomyopathy or HF (Table 1).

## 3. Double-Blind Clinical Trials of First-Generation Cell-Based Therapies

### 3.1. Unfractionated BM-Derived Mononuclear Cells

The BM is a source of a variety of multipotent precursors, including mononuclear cells (MNCs), hematopoietic stem cells, endothelial progenitor cells, and mesenchymal stem cells (MSCs). BM-derived MNCs (BM-MNCs) are relatively easy to harvest via BM biopsy/aspiration, and subsequently isolate via density gradient.

BM-MNCs can be collected from BM cells a few hours before administration, without the need to expand or culture cells. Although BM-MNCs can be collected for allogeneic use, the majority of clinical trials have minimized the risk of rejection and the need for immunosuppressants through the use of autologous BM-MNCs (Table 1).

Despite encouraging efficacy in small, open-label studies [58,59] and a few small (n = 20–50) double-blind studies demonstrating improvements in LVEF and LV volume at 6 or 12 months [12,16], the majority of larger, double-blind trials, such as **FOCUS-CCTRN** (N = 92) [18], **TAC-HFT** (N = 65) [22], and **MiHeart** (N = 160) [26], did not result in significant improvements in LVEF or LV volume (Table 1). Given the negative outcomes of double-blind clinical trials, testing of BM-MNCs has largely been abandoned.

### 3.2. Mesenchymal Stem Cells

MSCs are a subset of heterogeneous non-hematopoietic adult stem cells that express surface markers CD105, CD73, and CD90. Although they originate in the mesoderm, MSCs can self-renew and differentiate into cells of other lineages and not just those from the mesoderm. MSCs can be harvested from various tissues, including BM, adult adipose tissue, and UC, and they are relatively easy to isolate and then expand in vitro, although the cells will eventually senesce in culture. As with BM-MNCs, the transplantation of BM-derived MSCs (BM-MSCs) can be allogeneic or autologous.

BM-MSCs have been extensively studied in double-blind clinical trials in patients with AMI, HF, or ischemic cardiomyopathy. Most studies did not detect significant improvements in LVEF or LV volumes (**TAC-HFT** [22]; **CONCERT_CCRTN** [43]) or meet their composite primary endpoints (**CHART-1** [38]; **DREAM-HF** [40,41]). However, there were some indications of potential benefit with this approach in these and other trials. The **MSC-HF** trial in 60 patients with ischemic HF met its primary endpoint and showed a dose-response relationship with improvements in LVEF and LV end-systolic volume (LVESV), as well as improvements in QoL [34]. Improvements in QoL were also observed in the **CONCERT_CCRTN** trial [43]. Moreover, at the 4-year follow-up, the BM-MSC-treated patients experienced significantly fewer hospitalizations for angina [35]. The **TRIDENT** study assessed the effect of high doses of BM-MSCs (100 million) versus low doses (20 million) in 30 patients with ischemic cardiomyopathy, and it was noted that high doses improved LV function and New York Heart Association (NYHA) class versus lower doses [37]. Although the primary composite endpoint (all-cause mortality, worsening HF, Minnesota Living with Heart Failure Questionnaire [MLHFQ] score, 6-minute walk distance, LVESV, and LVEF) at 39 weeks did not improve in the **CHART-1** trial [38], a reduced risk of death or cardiovascular hospitalization was observed with longer-term follow-up in patients with LV end-diastolic volume of 200–370 mL [39]. Similarly, although BM-MSCs did not reduce the risk of the primary endpoint of time to recurrent non-fatal decompensated HF-related major adverse cardiovascular events (HF-MACE) in **DREAM-HF**, reductions in the risk of other clinical outcomes, such as myocardial infarction (MI) or stroke, were noted [41].

### 3.3. UC-Derived MSCs

UC-derived MSCs (UC-MSCs) offer advantages over BM-MSCs in that they are widely available, and do not require an invasive procedure to harvest. Moreover, they have low immunogenicity [60], and a higher proliferative capacity [61] than BM-MSCs. Clinical trials with UC-MSCs are limited in number, but are predominantly positive.

In **RIMECARD,** a randomized, double-blind trial of 30 patients with HFrEF, an intravenous (i.v.) infusion of allogeneic UC-MSCs (1 × 10^6^ cells/kg) was compared with placebo and shown to improve LVEF, NYHA functional class, and QoL (MLHFQ) [45]. In a trial studying the safety and efficacy of an intracoronary infusion of UC-MSCs (6 × 10^6^ cells/kg) in 116 patients with AMI, cell therapy was also shown to improve LVEF, myocardial viability, and decrease in LVESV and LVEDV compared with placebo at 18 months [44]. A double-blind clinical trial in 50 patients with LVEF ≤ 45% who were selected to receive an elective coronary artery bypass graft (CABG) assessed the safety and efficacy of UC-MSCs (1 × 10^8^ cells/kg), with or without administration with a bovine collagen hydrogel to aid engraftment and functional integration, with control patients not receiving UC-MSCs. At 12 months, mean infarct size as a percentage of LV mass decreased after treatment with UC-MSCs with collagen hydrogel, but increased with UC-MSCs alone or with no UC-MSCs [46]. This study suggests that supporting the engraftment of cells may provide additional benefits, and the molecular mechanism and retention of UC-MSCs in the heart should be clarified in the future.

### 3.4. Adipose-Derived Regenerative Cells/Adipose-Derived MSCs

Adipose-derived regenerative cells (ADRCs) are a heterogeneous population of multipotent cells, including MSCs, obtained from the vascular stromal fraction of adipose tissue [62]. The adipose-derived MSCs are more abundant than BM-MSCs and harvesting (via liposuction) is arguably less invasive than BM aspiration. Moreover, ADRCs do not require culture or expansion.

Preclinical trials have shown beneficial effects in animal models of ischemic cardiomyopathy [63,64,65], but the results from double-blind clinical trials are limited and mixed. The **PRECISE** trial examined the safety and feasibility of administering ADRCs in 27 patients with coronary artery disease not amenable to revascularization, and ADRC treatment, but not placebo treatment, was associated with a significant increase (*p* < 0.001) in LV total mass from baseline to 6 months. In addition, LV infarcted mass increased with placebo (*p* = 0.01) but not ADRC treatment. However, there were no significant changes in LVEF or LV volume with either treatment. The **PRECISE** trial was limited by a small sample size and imbalances in baseline magnetic resonance imaging (MRI), single-photon emission computed tomography (SPECT) measurements, and age between treatment groups [47].

The **ATHENA I and II** trials aimed to assess the effects of ADRCs in patients with chronic ischemic cardiomyopathy (LVEF ≥ 20% to ≤ 45%). Enrollment in these trials was terminated prematurely due to cerebrovascular events deemed unrelated to the cell product. In those patients who were enrolled, no improvements in LV function or volume were observed with ADRCs; however, an improvement in QoL (MLHFQ) was reported with ADRCs [48].

Two completed Phase 2 trials of allogeneic ARDCs have yet to publish their results: **SCIENCE** and **CSCC_ASCII** [56,57].

### 3.5. C-Kit-Positive Cardiac Cells

C-kit-positive cardiac cells (CPCs) are multipotent, clonogenic stem cells with subpopulations that can preferentially differentiate into myocytes or endothelial cells. Treatment with these cells has been shown to promote cardiac regeneration and angiogenesis via paracrine effects in animal models [66,67].

Several clinical trials have assessed the potential effects of CPCs in patients with HF. The **CONCERT_CCRTN** trial assessed the safety and efficacy of autologous CPCs, BM-MSCs, and a combination of BM-MSCs and CPCs, versus placebo in 125 patients with ischemic HF [43]. Interestingly, CPCs were noted to reduce HF-MACE over 12 months; however, improvements in LV function and reductions in scar size were not noted. Thus, the mechanism for the reduction in HF-MACE in the **CONCERT_CCRTN** trial is unclear. **SCIPIO**, a Phase 1 trial assessing the effect of CPCs in patients with post-infarction LV dysfunction before CABG, reported encouraging efficacy results, with increases in LVEF and decreases in scar size [68]. However, the publication was later retracted due to doubts over the reliability of the work performed by the laboratory that had prepared the cells [69].

### 3.6. Summary of First-Generation Cell Therapies

Preclinical studies and double-blind clinical trials have confirmed the safety of many first-generation stem cell therapies in patients with HF or ischemic cardiomyopathy. First-generation cell therapies, such as BM-MNCs, appear to have low engraftment rates, and functional benefits appear limited and mediated largely by paracrine effects supporting existing CMs, rather than an ability to integrate and regenerate new myocardium. Approaches that aid stem cell engraftment and promote functional integration, may therefore be required to observe consistent clinical benefits with these categories of cell therapies in patients with HF.

## 4. Next-Generation Stem Cell Therapies

For regenerative therapies to realize their potential in HF, the new cells must not only have paracrine effects, but also survive, engraft, create gap junctions, and couple electrically with native CMs. By using pluripotent stem cells (PSCs)—which can differentiate into all cell types—it is possible to culture LV-specific CMs [70]. PSC-derived cell products represent a new generation of cell therapies based on the transplantation of mature cell types that may be more likely to engraft and integrate electrically with the host myocardium compared with first-generation multipotent stem cell therapies.

There are two types of PSCs: embryonic stem cells (ESCs) and induced PSCs (iPSCs).

▪ ESCs are derived from the inner cell mass of blastocysts and can differentiate into all three embryonic germ layers.
▪ iPSCs are generated from fully differentiated adult somatic cells, e.g., skin fibroblasts or peripheral blood cells. Somatic cells are reprogrammed to become PSCs, usually by overexpressing the transcription factors required for pluripotency [71,72].

### 4.1. Human PSC-Derived CMs in Preclinical Models of HF

Studies of rodent models of myocardial injury have reported the beneficial effects of human ESC (hESC)-derived CMs (hESC-CMs). Injection of 1 × 10^6^ hESC-CMs into the hearts of immunocompromised mice following an MI induced by ligation of the left anterior descending (LAD) coronary artery, resulted in improvements in LV function at 4 weeks, although not at 12 weeks [73]. This short-term benefit is likely reflective of paracrine effects. Although hESC-CMs were shown to integrate and mature in vivo, it was suggested that graft size may have limited the longer-term functional benefit in this study. Another study has noted a longer-term benefit with an intramyocardial injection of 1 × 10^6^ hESC-CMs in mice after induction of an MI: LVEF was improved at Day 28 and Day 60, scar size and CM apoptosis were significantly reduced, and CM proliferation, capillary bed, and arteriole number all increased [74]. hESC-CMs transplanted into a guinea pig model of cardiac injury have also been reported to improve the mechanical function of the heart and reduce ventricular tachycardia [75]. Moreover, grafts were heterogeneous, with uncoupled regions and regions that contracted synchronously with the host heart [75], suggesting a level of electromechanical integration, but also showing a need to further optimize engraftment. Similar to studies with hESC-CMs, human iPSC (hiPSC)-derived CMs (hiPSC-CMs) have also shown some benefits in rodent models. An intramyocardial injection of 10 × 10^6^ hiPSC-CMs into the myocardium 10 days after ligation of the LAD coronary artery, resulted in reduced mortality and cardiac remodeling versus controls, and LVEF increased by almost 20% after 4 weeks [76]. Grafted CMs could also be detected 1 month after transplantation in this study. In a rat model of HF, a tissue-engineered patch embedded with hiPSC-CMs and human neonatal fibroblasts was grafted onto the epicardial surface covering the infarcted tissue, and electrical activity was found to be improved and end-diastolic pressure reduced after 3 weeks [77].

### 4.2. Human Pluripotent Stem Cell Cardiomyocytes in Large Animal Models of HF

It is notable, however, that rodent hearts show marked differences in anatomy and physiology compared with human hearts, such as a much faster heart rate. Consequently, cell-based therapies should also be tested in large animal models to provide a better indication of efficacy and safety. In a porcine model of AMI, intramyocardial injections of three cell types derived from hiPSCs (CMs, endothelial cells, and smooth muscle cells) were administered through an epicardial fibrin patch loaded with insulin growth factor 1 to promote survival. This approach was shown to result in engraftment and improved LV function after 4 weeks, without inducing ventricular arrhythmias [78]. Another porcine model of MI has demonstrated stable engraftment that formed vascular networks and resulted in a large degree of remuscularization in the heart after transplantation with hESC-CMs. Although no teratomas were observed in that study, ventricular tachyarrhythmias were observed [79]. Studies in non-human primates have also produced promising findings. When 1 billion hESC-CMs were injected into the myocardium of immunosuppressed macaques 2 weeks after induction of an MI, significant remuscularization of the infarcted myocardium was noted. Grafts were shown to have developed electromechanical junctions and showed synchronization of calcium transients to the electrocardiogram from the host myocardium, indicating electromechanical coupling. In contrast to small animal models, however, non-fatal ventricular arrhythmias were also observed [80]. Another study of ~750 × 10^6^ hESC-CMs transplanted into a macaque monkey ischemia-reperfusion model of MI has demonstrated improvements in LVEF at 1 month and 3 months post-transplantation. Grafts were shown to have formed electromechanical junctions with the host myocardium, but a subset of animals were also noted to experience graft-associated ventricular arrhythmias [81]. In immunosuppressed cynomolgus monkeys, an intramyocardial injection of 4 × 10^8^ allogeneic iPSC-derived CMs (iPSC-CMs) 14 days after a 3-hour occlusion of the LAD coronary artery, resulted in improved contractile function at 4 and 12 weeks. Moreover, the grafts survived for 12 weeks and showed electrical coupling with the host CMs. This study reported an increased incidence of ventricular tachycardia with iPSC-CM treatment compared with vehicle-treated controls, but this was transient [82].

Preclinical studies in animal models have shown that hPSC-derived CMs (hPSC-CMs) can engraft into the host myocardium, grafts can be sustained over several months, and can achieve electromechanical coupling with the host myocardium and improve LV function. Although some of the benefits of hPSC-CMs may be due to paracrine mechanisms, the presence of myocardium remuscularization and electromechanical coupling indicate the potential for benefits due to direct interactions between hPSC-CMs and host CMs.

## 5. Challenges for hPSC-Based Regenerative Therapies in HF

For next-generation hPSC-based regenerative stem cell therapies to be tested and used in patients with HF, several concerns and challenges need to be addressed, including the potential risk of teratomas and arrhythmias, the need for an optimal delivery system and improved engraftment rates and survival, as well as large-scale production.

### 5.1. Teratoma Prevention

Teratomas are tumors made up of tissues from multiple germ layers. The ability of undifferentiated PSCs to form any cell type means that they form teratomas after transplantation [83,84]. Many preclinical studies have not observed teratoma formation following the administration of hPSC-CMs [75,76,80,81]. However, the true incidence of teratomas may be under-represented in some preclinical studies, which have often used relatively few animals and relatively short follow-up. Moreover, even a small risk may be clinically significant if millions of cells are injected. Indeed, undifferentiated hPSCs can give rise to teratomas even if only 0.025% of residual undifferentiated hPSCs remain [85]. There is therefore a need to develop technologies to aid early detection of teratomas, and it has been suggested that a combination of biomarkers (α-fetoprotein, carcinoembryonic antigen, and human chorionic gonadotrophin) along with an MRI, may provide a sensitive approach for identifying teratomas from hPSCs [86].

In addition to improved detection of teratomas with hPSCs, it is important to prevent teratoma formation through optimized pre-implantation protocols. One approach to limit the potential for teratomas with hPSCs, is to purify cultures to remove any undifferentiated cells before administration. Multiple approaches have been assessed to help identify and remove undifferentiated cells (Table 2). Use of a monoclonal antibody against cell surface antigens specific to hPSCs, can allow separation of cells through fluorescence-activated cell sorting [87]. However, cell sorting may be impractical when large numbers of cells are required. The use of small molecule inhibitors may also reduce the risk of teratomas, inducing the selective apoptosis of undifferentiated hPSCs [88]. For example, survivin is an anti-apoptotic factor specific to hPSCs, and chemical inhibitors of this factor, such as quercetin or YM155, have been reported to promote cell death in undifferentiated hPSCs, but not differentiated cells [89]. Treatment of in vitro cultures with brentuximab vedotin, which targets CD30-positive hiPSCs, has also been reported to promote cell death of non-differentiated hiPSCs and reduce teratoma formation in mice [90]. Another approach to eliminating undifferentiated hPSCs, is through metabolic selection (Table 2). Fatty acid synthesis is important for the survival of undifferentiated hiPSCs, but not hiPSC-CMs; consequently, inhibition of cells with fatty acid synthase before transplantation represents an approach for eliminating undifferentiated cells and minimizing the risk of teratomas [91]. Undifferentiated hPSCs use glutamine and glucose to produce energy, but cannot use lactate [92]. In contrast, hPSC-CMs can use lactate as an energy source. By culturing cells in a glucose- and glutamine-free medium supplemented with lactate, undifferentiated hPSCs can be eliminated to the level of <0.001% [88,92,93]. Glucose can inhibit maturation of hPSC-CMs [94], and therefore metabolic selection by restricting glucose may also aid the maturation of CMs during purification. Methionine is also required in large amounts by hPSCs, and prolonged depletion of methionine can lead to selective apoptosis of hPSCs [95]. Metabolic selection of hPSC-CMs from undifferentiated cells represents an approach that can be used on large-scale cultures and with limited requirements for specific or expensive compounds [88].

### 5.2. Risk Reduction of Arrhythmia after Transplantation

Electrical integration of the grafted cells into the myocardium is an important goal of regenerative cell therapy, and engraftment arrhythmias represent an obstacle to their use clinically. Previous studies have reported the development of ventricular arrhythmia after transplantation of hPSC-CMs into the hearts of larger animal and non-human primate models [79,80,82,107]. These arrhythmias typically occur within the first two to three weeks after transplantation of hPSC-CMs and then may persist/reappear for up to a month, after which the heightened risk for new events disappears [79]. A study using electrical mapping and pacing suggested that the mechanism of ventricular tachycardia after transplantation is automaticity rather than macro-reentry. Contamination of atrial cells, pacemaker cells, and non-ventricular CMs may cause arrhythmias [79]. Cell dose, injection volume, cell condition, and cell retention rate may also be important.

There are various potential strategies for the prevention of arrhythmias. Ensuring that transplanted cells are purified and do not include non-CM cells may aid electromechanical coupling. Moreover, transplanting hiPSC-CMs of a ventricular phenotype with electrophysiological characteristics close to those of the host tissue may also aid electrical integration. The initial hPSC-CMs were electrophysiologically immature. For example, the resting membrane potential is less hyperpolarized in immature hPSC-CMs (approximately −60 mV, similar to that of nodal cells) than in mature ventricular CMs (approximately −90 mV). Furthermore, immature hPSC-CMs express high levels of hyperpolarization-activated cyclic nucleotide-gated channel 4 in the plasma membrane, which is characteristic of pacemaker cells. These aspects make it easier for immature hPSC-CMs to beat spontaneously (i.e., to show automaticity), whereas adult ventricular CMs are electrically quiescent until triggered by the depolarization of adjacent cells [108]. Therefore, transplantation of more mature ventricular CMs may be useful for the reduction of arrhythmogenic risk. Studies have shown that differentiation and purification protocols can produce CMs of the ventricular phenotype for transplantation [109]. Ensuring a high survival rate of grafts is also important as necrotic tissue may cause inflammation and serve as a substrate for arrhythmias.

When large numbers of hiPSC-CMs were transplanted, single floating frozen cells were commonly used. These cells were thawed just before usage, but the cell surface proteins (ion channels, growth factor receptors, cell adhesion molecules, etc.) of these cells were destroyed by enzyme digestion, and the freeze-thaw process impaired cell survival after transplantation. As a result, the cell engraftment rate became extremely low, and the dead cells had the potential to cause local inflammation and injury to the surviving CMs or host CMs, resulting in induction of automaticity arrhythmia. Transplantation of a large volume of hiPSC-CMs at one site is not desirable, as it may destroy the physiological electrical conduction system of the host CMs. Finally, myocardial damage from intramyocardial injections could also trigger arrhythmias. Intramyocardial transplant injection devices that efficiently and safely introduce and distribute hiPSC-CM aggregates/spheroids, which have been reported to improve cell survival, engraftment, and cardiac function in rodents and pigs versus suspensions of single cells [99,107] are also in development [110]. We deduced that the improvement in transplantation techniques may greatly reduce graft-induced arrhythmia. Another approach to minimize the impact of engraftment arrhythmia is to employ pharmacologic approaches. Indeed, ivabradine and amiodarone have been used to effectively suppress engraftment arrhythmia in a porcine model of MI treated with hPSC-CMs [111].

### 5.3. Optimizing Delivery

**Figure 2:**
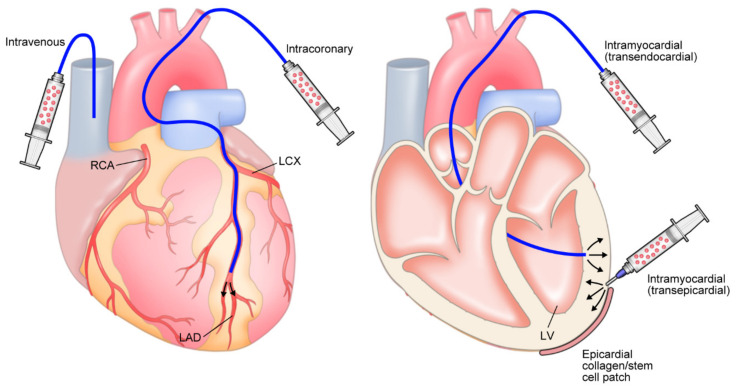
Overview of implantation methods and devices for engrafting hiPSC-CMs. hiPSC, human-induced pluripotent stem cell-derived cardiomyocyte; LAD, left anterior descending; LCX, left circumflex coronary artery; LV, left ventricle; RCA, right coronary artery.

There are numerous routes for administering hiPSC-CMs (Figure 2). The standard procedure for introducing cells into the heart is to inject them via intramyocardial (usually transendocardial or transepicardial) or intracoronary routes. Intracoronary delivery has the advantage of being less invasive than approaches requiring surgery, such as the placement of patches or transepicardial injections. Intracoronary injection may be unsuitable for delivering larger cells, such as MSCs, which could occlude the microcirculation and for use in patients with HF who have highly diseased arteries. A study in pigs has suggested that retention of peripheral blood MNCs is better after intramyocardial injection (11 ± 3%) than after intracoronary injection (2.6 ± 0.3%), with a smaller proportion of cells leaving the heart and entering the pulmonary circulation (intramyocardial injection, 26 ± 3%; intracoronary injection, 47 ± 1%) [112]. Indeed, it has been noted that 1 h after intracoronary injection, only 2–5% of cells were detected in the heart, with the majority found in the liver and spleen [113]. Graft survival is also poor following intracoronary administration of dispersed hPSC-CMs [114]. Although intracoronary injection of hPSC-CM aggregates can lead to partial engraftment, cardiac ischemia can develop and result in scars similar in size to the injected spheroids [114]. Another challenge for intracoronary delivery, is that hPSC-CMs would need to migrate from the vasculature into the myocardium.

Intramyocardial injections offer several other advantages over intracoronary administration, such as the ability to target cells to the myocardium and to a specific location, and the delivery of larger cells or aggregates/cardiospheres that might otherwise occlude microvessels. However, specialist training may be required for intramyocardial injection, and there is potential for perforation and myocardial damage [115]. Most clinical studies have used intramyocardial delivery, usually in the form of transendocardial catheter injections [115].

### 5.4. Further Improvement in Engraftment Rates and Longevity

Engraftment rates with hPSC-CMs still remain relatively low (e.g., no grafted hESC-CMs could be detected 4 months after administration of a fibrin patch loaded with hESC-CMs in a rat model of HF [116] or 140 days after administration of a cell suspension in a cynomolgus monkey model of MI [117]). Therefore, there is a need to further improve engraftment. It has been suggested that engraftment could be improved through tissue engineering and alternative methods of transplantation [118]. 

#### 5.4.1. Cardiospheres

Suspensions of single stem cell-derived CMs tend to graft poorly. The formation of PSC-derived CM (PSC-CM) aggregates/spheroids through cell–cell adhesion has been reported to improve cell survival when injected into mouse hearts [99]. Intramyocardial injection of spheroids—made up of approximately 1000 hPSC-CMs—into the infarcted hearts of rodents and pigs, produced significantly better engraftment and greater improvements in cardiac function versus suspensions of single cells [107]. PSC-CM aggregates/spheroids were generated in a floating cell condition, which means that they do not require enzyme digestion for harvesting the cells; cell surface proteins (such as ion channels, growth factor receptors, cell adhesion molecules), as well as extracellular matrix and matrix-bound growth factors are intact, which in turn greatly improves cell retention after transplantation. Conventional needles have a beveled edge at the tip, to cut the tissues and microvessels at the injection site, resulting in bleeding and spheroid leakage. Injection of spheroids into the myocardium of pigs by a specially designed needle with a cone-shaped tip and multiple side holes (SEEDPLANTER^®^) resulted in reduced tissue damage and bleeding, and better retention of spheroids within the myocardium than use of a conventional needle [110].

Culturing hPSC-CMs as spheroids may also lead them to acquire a more mature phenotype, which could improve engrafting and electrical coupling with native CMs. Co-culturing hiPSC-CMs with endothelial cells, smooth muscle cells, and cardiac fibroblasts in a three-dimensional (3D) environment, yielded spheroids that contained all four cell types, and hiPSC-CMs have a more adult-like phenotype than those produced in two-dimensional (2D) cultures [119].

#### 5.4.2. Delivering Cells via Epicardial Patches/Sheets

An alternative approach for the transplant of iPSCs is to use tissue engineering to produce sheets of cells, or ‘patches’, with specific architecture mimicking the structure that biological tissues achieve via encapsulation of cells in an extracellular matrix. These patches can be attached to the epicardial surface of the heart with adhesives or sutures [118,120].

Patches may be developed on a scaffold of natural or synthetic materials. Patches of hiPSC-CMs developed on a fibrin scaffold have been shown to improve engraftment and LV function compared with a suspension of single cells when transplanted onto the ventricle in a porcine model of MI [121]. A scaffold of polylactic-co-glycolic acid, a synthetic polymeric material, has also been used to develop a patch of iPSC-CMs on a large scale, and this approach has been reported to improve LVEF in a porcine model of ischemic cardiomyopathy [122]. Mesh-structured engineered heart tissue patches made up of iPSC-CMs have also been reported to improve LV function and establish dose-dependent remuscularization of guinea pig hearts [123].

Delivery of iPSC-CMs via patches offers some advantages, in that surgeons can visually confirm attachment and positioning. Attachment of the patch may cause less damage than an intramyocardial injection, and the patch provides a structural environment that may promote engrafting. Patches also have potential disadvantages in that their application may be more invasive than catheter-based delivery. Moreover, the epicardium and pericardial adipose tissue on the ventricular free wall [118,124] may present barriers that interfere with the full integration of the cells into the host myocardium. Epicardial patches may also be separated from the host myocardium by scar tissue, which may hinder electrical coupling with host CMs. Although several studies have noted functional and electrical recovery after grafting of iPSC-CM cell sheets/patches [121,122,125], it was reported that hESC-cardiac tissue patches introduced into a rat model of HF were electromechanically active, but were not electrically coupled to the host CMs at 4 weeks. In contrast, cells introduced via intramyocardial injection were electrically coupled to the host [120].

Further options for improving engraftment may be to utilize a combination of approaches. The injection of hPSC-CMs into the myocardium, accompanied by placement of an MSC-loaded patch on the epicardium has been noted to improve cardiac repair in rats [126]. The MSC patch released paracrine factors that enhanced vascular regeneration, and also significantly improved the retention and engraftment of intramyocardial injected hiPSC-CMs. 

### 5.5. Economic Improvement of Production

**Figure 3:**
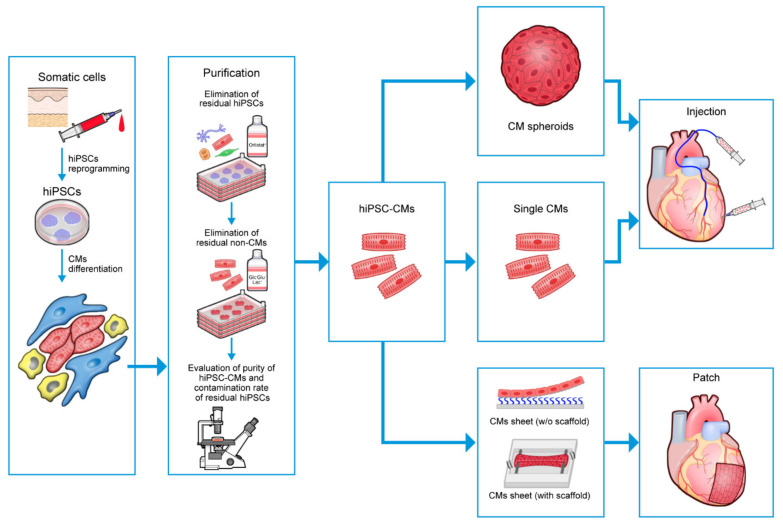
Overview of the scalable manufacturing of clinical-grade hiPSC-CMs. CM, cardiomyocyte; hiPSC, human-induced pluripotent stem cell; hiPSC-CM, human-induced pluripotent stem cell-derived cardiomyocyte; w/o, without.

It is estimated that approximately several hundred million to one billion CMs would be needed to completely replace the CMs lost in the LV of a patient with severe HFrEF [88]. Therefore, the production of hiPSC-CMs needs to be scalable to meet the demand to conduct trials and to treat patients if shown to be effective. Currently, initial culturing of hiPSCs and hiPSC-CMs can be performed efficiently and on a large scale using a 2D culture system [109], with cardiosphere development occurring in microwell plates after differentiation and purification [107] (Figure 3). It has been suggested, however, that 3D culture techniques may offer greater scalability, producing larger numbers of cells than traditional 2D cultures [88,127]. Moreover, 3D cultures allow iPSC-CMs to develop a more mature phenotype than 2D monolayers [119], possibly due to the low oxygen environment [128]. 3D suspension cultures may also be more economical, as there is no requirement to use expensive cell-adhesive coating proteins. Although massive 3D suspension culture systems offer the production of great numbers of hiPSC-CMs, there is a need to confirm the quality of hiPSC-CMs manufactured in the process, particularly in terms of the purity of CMs to minimize the risk of teratoma formation [129]. Metabolic purification systems that restrict glucose and glutamine and supplement lactate offer an approach that may allow the purification of hiPSC-CMs in massive 3D suspension culture systems.

## 6. Clinical Trials with hPSC-CMs

One small trial to assess the safety and feasibility of using hESC-derived cardiac progenitor cells (CPCs) to treat HF has already been completed: the **ESCORT** trial [70]. This trial assessed the efficacy of a fibrin patch embedded with hESC-derived CPCs implanted on the epicardium during CABG. Six patients with LVEF ≤ 35% and a history of MI were treated. No patients showed arrhythmias or developed teratomas during follow-up, but three patients showed clinically silent alloimmunization. At the 1-year follow-up, all patients assessed showed a reduction in HF symptoms. A significant increase in heart wall motion was also seen in cell-treated areas, along with a non-statistically significant increase in LVEF. 

Early phase trials to confirm the safety and efficacy of hPSC-CMs in HF are now ongoing (Table 3). These trials are relatively small (10–55 patients), with most being open-label and very few having a control arm. The trials are predominantly assessing the effect of hPSC-CMs in patients with ischemic HFrEF, although two studies also include patients with non-ischemic HFrEF. The number of transplanted cells varies, probably due to differences in cell purity, cell transplantation form, and engraftment rate. The primary objective of most of the trials is to assess safety. Assessment of LVEF or wall thickness by echocardiography are the primary objectives in only two studies; however, most other studies include echocardiography and MRI assessments of efficacy as secondary endpoints. Moreover, several studies have also included functional (6-minute walk distance/time) and QoL (MLHFQ) assessments.

Although preclinical trials have used both hESCs and hiPSCs, the current regulatory environment and potential ethical issues related to the use of hESCs means that the focus of most trials is on the use of hiPSCs (Table 3). Ongoing trials are also using allogeneic rather than autologous cells. There are several reasons why allogeneic cells may be preferred over autologous cells. The function of cells for autologous use in patients with HF may be compromised by age or comorbidities, or genetic disorders in the cases of some hypertrophic or dilated cardiomyopathies. In addition, allogeneic cells do not require harvesting, reprogramming, or quality checking for each host, and therefore, their production can occur more rapidly and on a larger scale than autologous cells. Autologous cells have some advantages over allogeneic cells in terms of improved engraftment and reduced risk of rejection, and the lack of requirements for immunosuppressants. The use of autologous cell therapy would thus be beneficial for patients with HF who are not tolerant of immunosuppressants. However, a more rapid and efficient process for obtaining, differentiating, and checking hiPSC-CMs from each patient would need to be established first.

## 7. Summary

First-generation cell-based therapies using multipotent cells demonstrated safety in preclinical models of HF, but poor engraftment rates and a limited ability to couple electrically with existing CMs meant that improvements in cardiac function in clinical trials were largely limited to those attributable to paracrine effects. Next-generation stem cell therapies using CMs derived from hESCs or, increasingly, from iPSCs, are in development and have shown the ability to engraft more successfully, and to improve electromechanical function of the heart in preclinical studies, including in non-human primates. These next-generation therapies are being enhanced by advances in techniques to improve engraftment rate and to minimize the risk of teratomas by purifying cells on a large scale. Clinical trials of allogeneic hiPSC-CMs in HF are now ongoing, providing hope for vast numbers of patients with few other options available.

## Figures and Tables

**Table 1:** Double-blind clinical trials of first-generation cell-based therapies for the treatment of HF.

							Key Findings			
Study Patient Population	Cell Type (Number)	Auto/ Allo	Phase	n	Follow-Up	Delivery Route	LVEF LV Volumes	Infarct/ Scar Size	QoL	Other
**BM-MNCs**										
Ruan 2005 [12] MI and LAD occlusion	BM-MNCs (not specified)	Auto	?	20	6 months	IC	Improved (BM-MNCs, 53.37–59.33%; control, 53.51–50.30%) Improved	–	–	–
Janssens 2006 [13] NCT00264316 STEMI and PCI	BM-MNCs (304 × 10^6^ nucleated cells, 172 × 10^6^ MNCs)	Auto	?	77	4 months	IC	ns (BM-MNCs, 48.5–51.8%; placebo, 46.9–49.1%)	–	–	–
Assmus 2009 [14] NCT00279175 STEMI with successful stent and LVEF ≤ 45%	BM-PCs ^1^	Auto	?	204	2 years	IC	ns (BM-MNCs, 46.5–53.7%; placebo, 40.4–46.8% at 2 years) ns	–	–	Improvement in composite primary endpoint vs. placebo (death, MI, or need for revascularization)
Traverse 2010 [15] STEMI with successful stent/angioplasty and LVEF ≤ 50%	BM-MNCs (100 × 10^6^ cells)	Auto	1	40	1 year	IC	ns (BM-MNCs, 49.0–55.2%; placebo, 48.6–57.0% at 6 months) ns	–	–	–
Hu 2011 [16] CHF due to severe ischemic cardiomyopathy (LVEF < 30%)	BM-MNCs (100 × 10^6^ cells)	Auto	?	60	6 months	IC	Improved (BM-MNCs, 22.78–33.80%; placebo, 24.95–31.82%) Improved	–	–	6MWT improved Reduction in BNP
**ASTAMI** Beitnes 2011 [17] Anterior STEMI and PCI	BM-MNCs (median: 68 × 10^6^ cells)	Auto	?	100	3 years	IC	ns (BM-MNCs, 45.7–47.5%; placebo, 46.9–46.8%) ns	–	–	–
**FOCUS-CCTRN** Perin 2012 [18] NCT00824005 HF (NYHA class II–III or CCS class II–IV) and LVEF ≤ 45%	BM-MNCs (100 × 10^6^ cells)	Auto	2	92	6 months	TE	ns (BM-MNCs, +1.4% from baseline; placebo, −1.3% from baseline) ns	ns	–	Maximum O_2_ consumption ns NT-proBNP ns
**SCAMI** Wohrle 2013 [19] Wohrle 2010 [20] MI and PCI conducted 6–48 h after symptoms	BM-MNCs (median: 324 × 10^6^ cells)	Auto	?	42	3 years 6 months	IC	ns (BM-MNCs, 53.5–54.0%; placebo, 55.7–59.4% at 3 years) ns	ns	–	–
Lu 2013 [21] Chronic MI (≥3 months), LVEF ≤ 35%, admitted for elective CABG	BM-MNCs (‘average’: 133.8 × 10^6^ cells)	Auto	?	50	12 months	IC	Improved (BM-MNCs, +13.5%; control, +8.0%) –	ns	–	–
**TAC-HFT** Heldman 2014 [22] NCT00768066 Ischemic cardiomyopathy and LVEF < 50%	BM-MNCs (CardiAMP^®^)	Auto	1/2	65	12 months	TE	ns (no change in LVEF) ns	ns	Improved	Functional capacity ns
Patila 2014 [23] NCT00418418 HF (NYHA class II–IV; LVEF 15–45%) and scheduled for CABG	BM-MNCs (median: 840 × 10^6^ cells)	Auto	?	104	12 months	IMI	ns (BM-MNCs, +4.8%; control, +5.6%) ns	–	–	NT-proBNP ns Myocardial viability ns
Hu 2015 [24] NCT01234181 STEMI and PCI and LV wall motion abnormality	Hypoxia pre-conditioned BM-MNCs (100 × 10^6^ cells)	Auto	1	36	12 months	IC	ns (normoxia BM-MNCs, 56.9–56.8%; hypoxia BM-MNCs, 50.9–56.1%; control, 57.1–59.6%) Improved	–	–	Pre-conditioned cells superior to non-pre-conditioned
**REGENERATE-AMI** Choudry 2016 [25] NCT00765453 STEMI and regional wall motion abnormality	BM-MNCs (mean: 59.8 × 10^6^ cells)	Auto	2	100	12 months	IC	ns (BM-MNCs, +5.1%; placebo, +2.8%) –	ns	ns	NYHA class ns Myocardial salvage index improved NT-proBNP decreased in both groups
**Mi-Heart** Martino 2015 [26] NCT00333827 Non-ischemic dilated cardiomyopathy (LVEF < 35%)	BM-MNCs (mean: 236 × 10^6^ cells)	Auto	?	160	12 months	IC	ns (BM-MNCs, 24.0–19.9%; placebo, 24.3–22.1%) ns	–	ns	– BNP ns
**BOOST-2** Wollert 2017 [27] STEMI and reduced LVEF Subgroup analysis of patients with S-CMR Seitz 2020 [28] ISRCTN17457407	BM-MNCs (mean: high 2060 × 10^6^ cells; low 700 × 10^6^ cells)	Auto	?	153 51	6 months	IC	ns (high BM-MNCs, +4.3%; low BM-MNCs, +3.8%; control, +3.3%) ns	–	–	– BM-MNCs did not enhance infarct perfusion
**TIME** Traverse 2012 [29] STEMI and PCI (LVEF ≤45%) Follow-up analysis Traverse 2018 [30] NCT00684021	BM-MNCs (150 × 10^6^ cells)	Auto	?	120	6 months 2 years	IC	ns (BM-MNCs, 45.2–48.3%; placebo, 44.5–47.8%) ns ns (BM-MNCs, +2.8%; placebo, +4.7%) Increase in LVEDVI with BM-MNCs	– –	– –	– –
Nicolau 2018 [31] STEMI and angioplasty (LVEF ≤ 50%)	BM-MNCs (100 × 10^6^ cells)	Auto	?	121	6 months	IC	ns (BM-MNCs, 44.63–44.74%; placebo, 42.23–43.50%) ns	ns	–	–
**COMPARE-CPM-RMI** Naseri 2018 [32] NCT01167751 STEMI (LVEF 20–45%)	BM-MNCs (mean: 564.63 × 10^6^ cells)	Auto	2/3	77	6 months 18 months	IMI	Improved (BM-MNCs, +7% vs. placebo; CD133+ cells, +9% vs. placebo) –	–	–	BM-MNCs were inferior to CD133+ cells
**BM-MSCs**										
Hare 2009 [33] MI and LVEF 30–60%	BM-MSCs (0.5, 1.6, 6 × 10^6^ cells/kg)	Allo	?	53	6 months	i.v.	ns (BM-MNCs, 50.4–56.9%; placebo, 48.7–56.1%) ns	–	–	6MWT ns Global symptom score improved
**TAC-HFT** Heldman 2014 [22] NCT00768066 Ischemic cardiomyopathy (LVEF < 50%)	BM-MSCs (not specified)	Auto	1/2	65	12 months	TE	ns (no change in LVEF) ns	Reduced	Improved	6MWT improved Regional myocardial function improved
**MSC-HF** Mathiasen 2015 [34] Mathiasen 2020 [35] NCT00644410 Severe ischemic HF (NYHA class II–III; LVEF < 45%)	BM-MSCs (mean: 77.5 × 10^6^ cells)	Auto	2	60	6 months 12 months 4 years	IMI	Improved (+6.2% vs. placebo at 6 and 12 months) LVESV reduced by 13 mL (6 months) and 17 mL (12 months) vs. placebo	ns	–	6MWT ns NYHA class ns 4 years: hospitalizations for angina reduced
Chullikana 2015 [36] AMI and PCI NCT00883727	BM-MSCs (4.0 × 10^6^ cells)	Allo	1/2	20	2 years	i.v.	ns (BM-MSCs 43.06–47.80%; placebo, 43.44–45.33%) –	ns	–	–
**TRIDENT** Florea 2017 [37] NCT02013674 Ischemic cardiomyopathy secondary to MI (LVEF ≤ 50%)	BM-MSCs (low [20 × 10^6^ cells] vs. high dose [100 × 10^6^ cells])	Allo	2	30	12 months	TE	Improved with high dose by 3.7 units –	Reduced	–	NYHA class improved NT-proBNP increased with low dose
**CHART-1** Bartunek 2017 [38] Follow-up: Bartunek 2020 [39] NCT01768702 Symptomatic ischemic HF (LVEF ≤ 35%)	Cardiopoietic BM-MSCs (24 × 10^6^ cells)	Auto	3	315	39 weeks 104 weeks	TE	– –	–	–	ns for composite primary endpoint Subgroup analysis suggests a beneficial effect in patients with low LVEDV 2-year follow-up confirmed benefits in patients with LV enlargement
**DREAM-HF** Borow 2019 [40] Perin 2023 [41] NCT02032004 Advanced stable chronic HFrEF	BM-MSCs (not specified)	Allo	3	565 (537 treated)	Median ~30 months	TE	?	?	?	Did not meet primary endpoint 58% reduction in MI or stroke 28% reduction in 3-point MACE
**COMPARE-AMI** Haddad 2020 [42] STEMI and LV dysfunction after PCI	CD133+ enriched BM-MSCs 10 × 10^6^ cells (one patient was injected with 5.2 × 10^6^ cells)	Allo	2	38	10 years	IC	?	–	–	10-year event-free survival ns
**CONCERT_CCRTN** Bolli 2021 [43] HF caused by ischemic cardiomyopathy (NYHA class I–III; LVEF ≤ 40%; scar ≥ 5% LV volume)	BM-MSCs ± CPCs (BM-MSCs, 150 × 10^6^ cells^;^ CPCs, 5 × 10^6^ cells)	Auto	2	125	12 months	TE	ns ns (BM-MSCs + CPCs, 29.21–29.91%; CPCs 26.31–26.96%; BM-MSCs, 29.26–31.12%; placebo, 29.66–29.35%)	–	Improved with MSCs + CPCs and with MSCs alone	6MWT ns Peak O_2_ consumption ns MACE decreased with CPCs NT-proBNP ns
**UC-MSCs**										
**Gao 2015 [44]** UC-MSCs STEMI and successful stent	UC-MSCs (6 × 10^6^ cells)	Allo	?	116	18 months	IC	Improved (UC-MSCs, +7.8%, placebo, 2.8%) Improved	–	–	Increase in myocardial viability with UC-MSCs
**RIMECARD** Bartolucci 2017 [45] NCT01739777 HFrEF (NYHA class I–III; LVEF ≤ 40%)	UC-MSCs (1 × 10^6^ cells/kg)	Allo	1/2	30	12 months	i.v.	Improved (TTE LVEF: UC-MSCs, 33.50–40.57%; placebo, 31.53–33.39%; CMR LVEF: UC-MSCs, 32.64–37.43%; placebo, 29.62–31.31%) ns	–	Improved	NYHA class improved Decreased BNP
He 2020 [46] NCT02635464 Chronic ischemic heart disease (LVEF ≤ 45%) requiring CABG	UC-MSCs in collagen hydrogel (100 × 10^6^ cells)	Allo	1	50	12 months	IMI	– –	Reduced	–	–
**ADRCs**										
**PRECISE** Perin 2014 [47] NCT00426868 Ischemic cardiomyopathy (NYHA class II–III or CCS class II–IV; LVEF ≤ 35%) not amenable to revascularization	ADRCs (0.4, 0.8, 1.2 × 10^6^ cells/kg) (mean: 42 × 10^6^ cells)	Auto	1	27	36 months	TE	ns ns	–	–	VO_2_ max ns
**ATHENA I and II** Henry 2017 [48] Multivessel CAD (NYHA class II–III or CCS class II–IV; LVEF 20–45%) not amenable to revascularization **DISCONTINUED**	ADRCs (**ATHENA I**, 40 × 10^6^ cells; **ATHENA II**, 80 × 10^6^ cells)	Auto	?	28 3	12 months	IMI	– –	–	Enrolment terminated prematurely due to non-ADRC-related AEs	
**Myoblasts**										
**MAGIC** Menasche 2008 [49] Ischemic cardiomyopathy (LVEF 15–35%) and indication for CABG	Myoblasts (low dose, 400 × 10^6^ cells; high dose, 800 × 10^6^ cells)	Auto	1	97	6 months	IMI	ns (low dose, +3.4%; high dose, +5.2%; placebo, +4.4%) Improved	ns	–	–
**MARVEL** Povsic 2011 [50] HF (NYHA class II–IV; LVEF < 35%) **DISCONTINUED**	Skeletal myoblasts (400 × 10^6^ cells or 800 × 10^6^ cells)	Auto	2b/3	23	6 months	IMI	– –	–	Discontinued for financial reasons following enrolment of 23 out of 330 planned patients Larger BNP increases with placebo vs. myoblast treatment	
**ALLSTAR** Makkar 2020 [51] Post-MI LV dysfunction (NYHA class II–IV; LVEF ≤ 45%; LV scar ≥15% LV mass) **DISCONTINUED**	CDCs (25 × 10^6^ cells)	Allo	?	142	Interim analysis at 6 months	IC	– Improved	ns	NT-proBNP reduced Discontinued based on prespecified interim analysis at 6 months that indicated futility with respect to primary endpoint	
**CAREMI** Fernandez-Aviles 2018 [52] STEMI and LVEF ≤ 45% and infarct > 25% LV mass	CSCs (35 × 10^6^ cells)	Allo	1/2	49	12 months	IC	ns (CSCs, +7.7%; placebo, +8.6%) ns	ns	–	NT-proBNP changes ns
**Ongoing trials/trials with results awaited**										
**CardiAMP^®^** **Biocardia** [53] Raval 2021 [54] Johnston 2022 [55] NCT02438306 Chronic LV dysfunction (NYHA class II–III; LVEF 20–40%) secondary to MI	BM-MNCs (not specified)	Auto	3	250	2 years	CardiAMP^®^ cell therapy system	1o: composite ^2^ 2o: survival, MACE, QoL	Estimated completion December 2024 Open-label, roll-in cohort (n = 10): 12 months: trend improvement in LVEF, 6MWT, QoL, NYHA 2 years: 100% survival; improved 6MWT and LVEF vs. baseline		
**SCIENCE** Paitazoglou 2019 [56] NCT02673164 Chronic ischemic HF (NYHA class II–III; LVEF < 45%)	ADRCs ^3^ (100 × 10^6^ cells)	Allo	2	133	12 months	TE	1o: LVESV 2o: SAEs	Completed December 2020		
**CSCC_ASCII** [57] NCT03092284 Chronic stable ischemic heart disease (NYHA class II–III; LVEF ≤ 45%)	AD-MSCs (100 × 10^6^ cells)	Allo	2	81	12 months	TE	1o: LVESV 2o: TEAEs, LVEF, KCCQ, Seattle Angina Questionnaire; 6MWT	Completed July 2022		

^1^ Progenitor cells; ^2^ Composite endpoint based on a three-tiered hierarchical analysis, including (i) all-cause death, (ii) non-fatal MACE events, (iii) change in 6MWT performance; ^3^ Cardiology Stem Cell Centre adipose-derived stromal cell. ?, uncertain/unidentified; –, not measured/reported; 6MWT, 6-minute walk test; AD-MSC, adipose-derived mesenchymal stem cell; ADRC, adipose-derived regenerative cell; AE, adverse event; allo, allogeneic; auto, autologous; BM-MNC, bone marrow-derived mononuclear cell; BM-PC, bone marrow-derived progenitor cell; BNP, B-type natriuretic peptide; CABG, coronary artery bypass graft; CAD, coronary artery disease; CCS, Canadian Cardiovascular Society; CDC, cardiosphere-derived cell; CHF, congestive heart failure; CMR, cardiac magnetic resonance; CPC, c-kit-positive cardiac cell; CSC, cardiac stem cells; HF, heart failure; HFrEF, heart failure with reduced ejection fraction; IC, intracoronary; IMI, intramyocardial injection; i.v. intravenous; KCCQ, Kansas City Cardiomyopathy Questionnaire; LAD, left anterior descending; LV, left ventricular; LVEDV, left ventricular end-diastolic volume; LVEDVI, left ventricular end-diastolic volume index; LVEF, left ventricular ejection fraction; LVESV, left ventricular end-systolic volume; MACE, major adverse cardiovascular event; MI, myocardial infarction; MNC, mononuclear cell; MSC, mesenchymal stem cell; ns, not statistically significant relative to comparator; NT-proBNP, N-terminal pro B-type natriuretic peptide; NYHA, New York Heart Association; O_2_, oxygen; PCI, percutaneous coronary intervention; QoL, quality of life; s-CMR, stress perfusion magnetic resonance imaging; SAE, serious adverse event; STEMI, ST segment elevation myocardial infarction; TE, transendocardial; TEAE, treatment-emergent adverse event; TTE, transthoracic echocardiogram; UC-MSC, umbilical cord-derived mesenchymal stem cell; VO_2_ max, maximal oxygen consumption.

**Table 2:** Approaches for purifying cardiomyocyte cultures (adapted from Soma et al. [88]).

Approach	Mechanism	Advantages	Disadvantages
**Cell sorting using MACS or FACS**			
Lectins [96]	hPSC-specific biomarker (lectin) mediated cell separation by MACS	Simple Accurate	Requires cell dissociation Scalability due to labor-intensive process
SSEA-5 [87]	Antibody targeting hPSC-specific cell surface H type-1 glycan and cells separated by FACS	Simple Accurate	Requires cell dissociation Scalability due to a labor-intensive process
TRA-1 60, SSEA-4 [97]	Antibody targeting hESC-specific cell surface H type-1 glycan and cells separated by MACS and FACS	Simple Accurate	Requires cell dissociation Scalability due to a labor-intensive process
SIRPA [98]	hPSC-CM-specific markers	Simple Accurate Selective for hPSC-CMs	
Mitochondria [99]	Differences in mitochondrial number identified by accumulation of fluorescent mitochondrion-specific dye in CMs	Simple Accurate	
**Metabolic selection**			
Glucose/glutamine depletion [92,100]	CMs, but not undifferentiated hPSCs, can utilize lactate to generate energy in the absence of glucose and glutamine. Incubation of cells in glucose- and glutamine-free media supplemented with lactate results in elimination of undifferentiated cells	Cell dissociation not required Can be used on large-scale cultures Compounds are cheap and readily available Does not require specific compounds Selective for hSPC-CMs	Approach cannot be used for other hPSC-derivatives
Methionine depletion [95]	hPSCs require high amounts of methionine. Prolonged methionine depletion induced apoptosis of hPSCs	Does not require specific compounds	Concern about effects on hPSC-derived differentiated cells
PluriSIns [101]	Pluripotent cell-specific inhibitor of stearoyl-coA desaturase, a key enzyme in oleate synthesis, which induces apoptosis of hPSCs	Does not require cell dissociation	
Fatty acid synthase inhibition [91]	Undifferentiated hPSCs express different fatty acid biosynthesis enzymes to differentiated cells Inhibition of fatty acid synthase reduces phosphatidylcholine, a key metabolite for survival, inducing apoptosis of hPSCs, but not hPSC-derived cells, including CMs	Can be used on large scale cultures Cost effective Can be used for a variety of differentiated cells	
**Addition of compounds**			
Inhibitors of survivin [89]	Inhibition of hPSC-specific antiapoptotic factor	Applicable to large scale culture Rapid	
D-3 [102]	A phospho-D peptide that causes cell death when dephosphorylated by alkaline phosphatases, which are overexpressed on hPSCs, but not hPSC-CMs	Does not require dissociation	Concern about effects on hPSC-derived differentiated cells
Lectin-toxin fusion protein [103]	Binds to hPSCs only and delivers cytotoxic protein when internalized, eliminating hPSCs		
Clostridium perfringens enterotoxin [104]	Toxic that binds to Claudin-6, a tight-junction protein specific to hPSCs, and kills undifferentiated cells		
**Other**			
Glypican-3 [105]	Pluripotent-state specific immunogenic antigen targeted by glypican-3-reactive cytotoxic T lymphocytes	Application to vaccinations and T-cell therapy targeting GPC3	Incomplete elimination of hPSCs
Brentuximab vedotin [90]	Antibody-drug conjugate targeting CD30, a cell surface antigen expressed specifically on hiPSCs		
MicroRNA-302a-5p [106]	MicroRNA-302a-5p is highly expressed in hPSCs, but not differentiated cells microRNA switch hPSC elimination system using miR-302a switch for controlling puromycin resistance before adding puromycin to kill undifferentiated cells	Application to Investigating dynamics based on intracellular information	Complex

CM, cardiomyocyte; FACS, fluorescence-activated cell sorting; hESC, human embryonic stem cell; hiPSC, human-induced pluripotent stem cell; hPSC, human pluripotent stem cell; hPSC-CM, human pluripotent stem cell-derived cardiomyocyte; MACS, magnetic-activated cell sorting.

**Table 3:** Ongoing clinical trials of hPSC-CM-derived therapies in HF.

ClinicalTrials.gov ID Location Phase	Participants	Cells	Duration	Doses	Delivery	Endpoints	Estimated Study Completion	Status
NCT04945018 **LAPiS** [130] Japan Phase 1/2 Open-label	10 patients with severe ischemic HFrEF (LVEF ≤ 40%) secondary to IHD	Allogeneic hiPSC-CM spheroids (HS-001)	12 months	‘Low dose (50 million)’ vs. ‘high dose (150 million)’	Injection using needle ‘SEEDPLANTER^®^’	1o: safety and tolerability (26 weeks) 2o: LVEF (Echo/MRI); myocardial wall motion; myocardial blood flow and viability (SPECT); 6MWT; KCCQ; EQ-5D-5L; NT-proBNP	March 2024	Recruiting
NCT04982081 [131] China Phase 1 Randomized double-blind parallel group	20 patients with severe congestive HFrEF (LVEF < 40%, both ischemic and non-ischemic)	Allogeneic hiPSC-CMs (HiCM-188)	12 months	100 × 10^6^ (n = 10) or 400 × 10^6^ (n = 10) cells	Catheter-based EC injection	1o: major SAEs ^1^ 2o: arrhythmias; tumors; immunogenicity; LV systolic function (Echo/MRI); 6MWT; NYHA; MLHFQ	July 2023	Recruiting
NCT05566600 [132] China Phase 1 Open-label	32 patients with worsening chronic ischemic HFrEF (LVEF < 40%, ischemic)	Allogeneic hiPSC-CMs in patients undergoing CABG	12 months	100, 200, or 400 × 10^6^ cells with CABG, or CABG only	Epicardial injection during CABG	1o: safety 2o: AEs; Holter monitoring; tumors; immunogenicity; LV systolic function (Echo/MRI); 6MWT; NYHA; MLHFQ; hospitalization for HF	July 2025	Not yet recruiting
NCT03763136 **HEAL-CHF** [133] China Phase 1/2 Randomized double-blind	20 patients with chronic LV dysfunction (LVEF ≥ 20% and ≤ 45%)	Allogeneic hPSC-CM	12 months	200 × 10^6^ cells in 2.5–5 mL medium suspension with CABG, or CABG only	Injection during CABG	1o: sustained ventricular arrhythmias; tumors 2o: overall left ventricular systolic performance; 6MWT; NYHA; MLHFQ; MACE; SAEs; penal reactive antibodies; donor-specific antibodies; severe arrhythmia; NT-proBNP	July 2023	Recruiting
NCT04696328 [134] Japan Phase 1 Open-label	10 patients with ischemic cardiomyopathy (LVEF ≤ 35%)	Allogeneic hiPSC-CM sheet	12 months	NR		1o: LVEF (Echo); safety 2o: number of responders; LV contraction; LV remodeling; NYHA; SAS; MLHFQ; SF-36; 6MWT; BNP; exercise tolerance; rejections	May 2023	Recruiting
NCT04396899 **BioVAT-HF** [135] Germany Phase 1/2 Open-label	53 patients with HFrEF (EF ≤ 35%, both ischemic and non-ischemic) with no realistic chance of a HT	BioVAT tissue: defined mixtures of hiPSC-CMs and stromal cells in a bovine collagen type 1 hydrogel	12 months	NA	Implantation on myocardium	1o: target heart wall thickness (Echo/MRI) and heart wall thickening fraction	October 2024	Recruiting
NCT05068674 **HECTOR** [136] USA Phase 1 Open-label	18 patients with chronic ischemic LV dysfunction (LVEF < 40%) secondary to MI treated with appropriate maximal medical therapy and a candidate for cardiac catheterization	Allogeneic hESC-CMs	36 months	50, 150, or 300 million cells spread over 10 injections	NR	1o: safety	October 2025	Recruiting

^1^ Composite of death, fatal MI, stroke, tamponade, cardiac perforation, ventricular arrhythmias affecting hemodynamics (>15 s), and tumorigenicity related to the hiPSC-CMs. 6MWT, 6-minute walk test; AE, adverse event; BioVAT, Biological Ventricular Assist Tissue; BNP, brain natriuretic peptide; CABG, coronary artery bypass grafting; EC, endocardial; Echo, echocardiography; EF, ejection fraction; EQ-5D-5L, EuroQol-5 Dimension-5 Level; hESC-CM, human embryonic stem cell-derived cardiomyocyte; HF, heart failure; HFrEF, heart failure with reduced ejection fraction; hiPSC, human-induced pluripotent stem cell; hiPSC-CM, human-induced pluripotent stem cell-derived cardiomyocyte; hPSC, human pluripotent stem cell; hPSC-CM, human pluripotent stem cell-derived cardiomyocyte; HT, heart transplantation; IHD, ischemic heart disease; KCCQ, Kansas City Cardiomyopathy Questionnaire; LV, left ventricular; LVEF, left ventricular ejection fraction; MACE, major adverse cardiovascular event; MI, myocardial infarction; MLHFQ, Minnesota Living with Heart Failure Questionnaire; MRI, magnetic resonance imaging; NA, not applicable; NR, not reported; NT-proBNP, N-terminal pro B-type natriuretic peptide; NYHA, New York Heart Association; SAE, serious adverse event; SAS, Specific Activity Scale; SF-36, 36-item Short Form Survey; SPECT, single-photon emission computed tomography.

## Data Availability

No new data were created or analyzed in this study. Data sharing is not applicable to this article.
